# Coastal road mortality of land crab during spawning migration

**DOI:** 10.1038/s41598-021-86143-z

**Published:** 2021-03-23

**Authors:** Mi Ryu, Jae Geun Kim

**Affiliations:** 1 Graduate School of Interdisciplinary Program in Environmental Education, Seoul National University, 1 Gwanak-ro, Gwanak-gu, Seoul, 08826 Korea; 2 Department of Biology Education, Seoul National University, 1 Gwanak-ro, Gwanak-gu, Seoul, 08826 Korea; 3 Center for Education Research, Seoul National University, 1 Gwanak-ro, Gwanak-gu, Seoul, 08826 Korea

**Keywords:** Animal migration, Conservation biology, Animal migration, Conservation biology

## Abstract

Land crabs are threatened by ocean sprawl even though they act as keystone consumers in coastal forest. Female land crabs must migrate to the sea annually to release larvae. However, they face the risk of road mortality which reduces ecological connectivity. We investigated the spawning migration rhythm and the roadkill of land crab. Migrating crabs and roadkilled crabs were recorded on coastal roads in South Korea from July 28 to August 27 in 2018. Female land crabs mainly released zoeae during spring tide. The number of roadkilled crabs also synchronized with migration peak. A majority (95%) of 739 roadkilled carcasses were female crabs. As a result, the female crabs accounted only 29.6% of the population which can lead to a population decline. The roadkill density was the highest in a residential area without cement guardrails. These results suggest the mitigation actions for land crab roadkill. Among them, prohibiting vehicular traffic between sunset and midnight during spring tides in the breeding season should increase the viability of the population.

## Introduction

Artificial structures around coastal regions have increased and caused “ocean sprawl” globally in the last few decades^1,2^. As the ocean sprawl intensifies, coastal developments restrict the movement between land and sea, and also reduce land-sea habitat connectivity^3^. Especially, they create barriers for animals and constrain the migration of vertebrates (e.g., turtles, seals). The physical barriers on the coastal shoreline also reduce the population of invertebrates including crustaceans and insects^4^, propagating negative effects for of shorebirds, fish, and small mammals^5^. These changes in coastal regions need to be addressed as threats to transition zones with significant biodiversity, such as mangroves^6^.

The rapid expansion of road networks has resulted in health, education, and leisure benefits^2^, and many local governments have constructed coastal roads to attract tourists by increasing access to coasts^7^. However, the need for forage and reproduction by wild animals remains^8^. The increased number of roads and traffic volume present serious physical obstacles and even death for many animal species^9–11^. Roadkill is the most direct impact of terrestrial fragmentation by roads^10,12^. Thus, roadkill has an obvious effect on the wildlife populations and ecosystems^2,13,14^. Roadkill studies have focused on a wide range of inland vertebrate species^10,15–17^. However, few studies have documented the roadkill of invertebrate groups in coastal regions^18–21^.

Land crabs (*Gecarcinus lateralis, Gecarcoidea natalis, Gecarcinus Ruricola, Ocypode quadrata*) contribute to the ecological connectivity between land and ocean and play an important role in both ecosystems^22^. As they burrow holes, they accelerate the erosion of stones, aerate and mix the soil and enrich and fertilize it by bringing organic materials^23^. Land crabs also act as cleaners by consuming carcasses and enable transfer of terrestrial resources to the sea through reproduction^24^. Land crabs also represent keystone consumers as controlling the dynamics of seedling recruitment and litter in coastal forests^25^. They are influenced strongly by environmental changes near the coastline. It is due to the fact that female land crabs must migrate to the coasts annually for reproduction^18^. Larvae of land crabs live in the sea, whereas adults have adapted to living on land^25^. Therefore, the ovigerous (egg-bearing) females release zoeae at the estuary or seashore. The zoeae then reach the sea where they attain maturity^18,26,27^. However, the coastal roads are barriers to this journey. In particular, female land crabs are likely to face roadkill during their breeding migration across the coastal roads.

The adult sex ratio is an important parameter in the population demographics of animals^28^. During reproduction, the number of females is relatively more important than the number of males for population growth and is potentially significant in conservation biology^29^. In particular, an increase in female roadkill during reproduction period can alters the sex ratio or demographic structure and increases the risk of local extinction^30,31^. Furthermore, the land crab roadkill does not involve merely adult female crabs but also tens of thousands of larvae for each ovigerous crab.

Roadkill varies depending on the animal lifecycle, seasons, and spatial characteristics^14,32,33^. Notably, the differences in road mortality are influenced by the movements associated with reproduction^30^. For example, amphibians experience frequent roadkill near breeding ponds during spring migration for spawning^34,35^. Land crabs are especially vulnerable because of the synchronized release to zoeae if there are coastal roads. Most of the land crabs migrate to release zoeae in the relation to the lunar phase (e.g., *Sesarma haematocheir*^36^; *Johngarthia lagostoma*^37^; *Ucides cordatus*^38^), which may affect the risk of roadkill. Therefore, it is essential to identify the key time and spots of roadkill for effective mitigation.

Here, we examined the land crab (*Sesarma haermatocheir*) roadkills during the zoea release migration. The objectives of this study are (1) to analyze the migration rhythm of land crabs releasing zoeae, the effect of traffic volume on roadkill, and the roadkill effect of environment on both sides of the coastal road; (2) to evaluate the sex-biased road mortality; and (3); to suggest mitigation actions.

## Result

### Migration rhythm of *Sesarma haematocheir*

**Figure 1:**
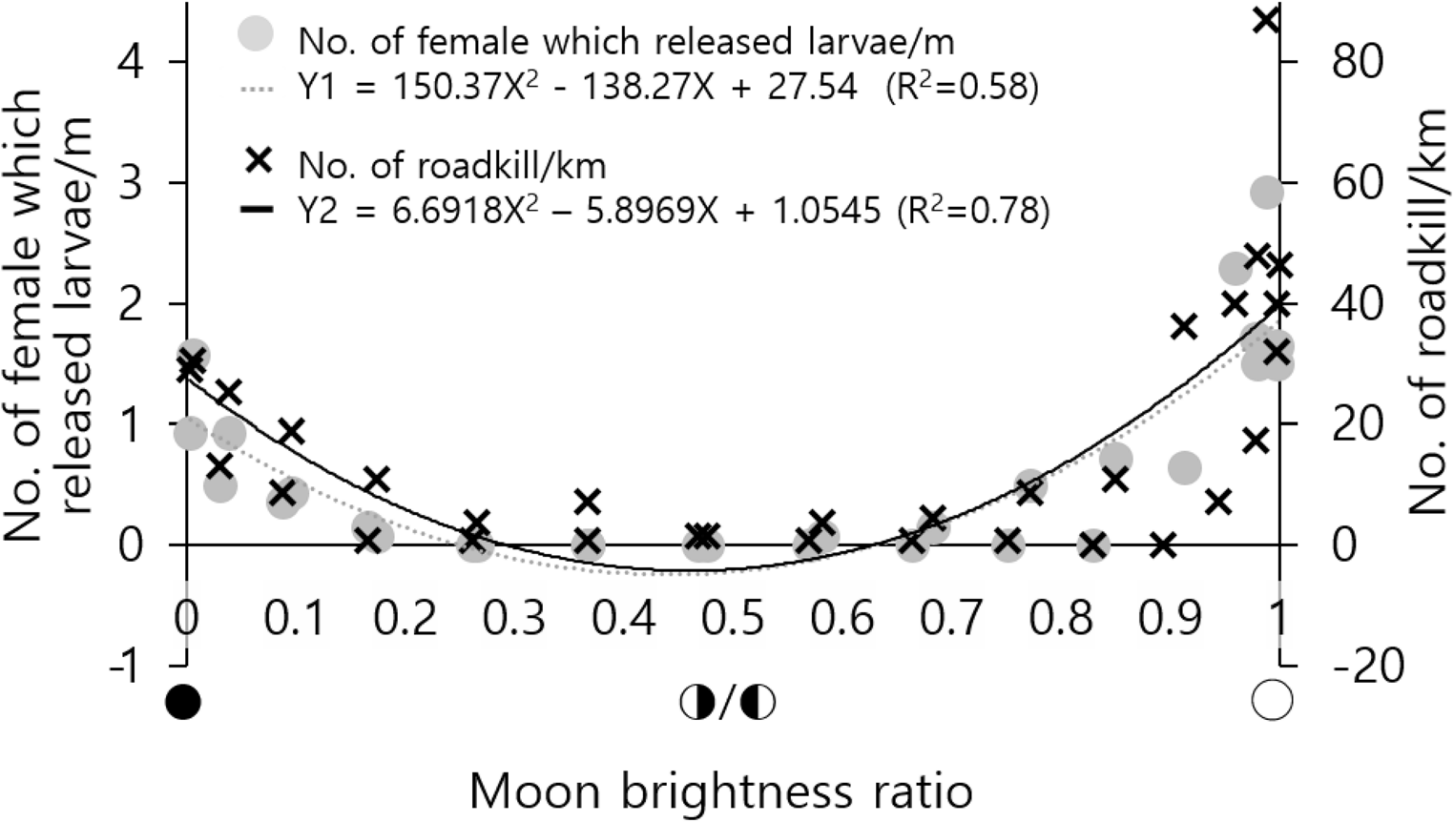
The density of zoeae release and roadkill depends on the moon brightness ratio. Estimated negative values from models are considered as 0.

In this study, 263 crabs were found to have crossed the 11 m-long section of the coastal road and gone down to the coast. Among them, 262 crabs were egg carrying females (99.6%). The number of land crabs that crossing the coastal road was higher during the spring tide at the new or full moon, and no crabs crossed the road during neap tide at the half-moon phase (Fig. 1).

After sunset, crabs started to migrate to shore. The number of crabs that moved from land to the sea peaked at high tide between 8:00 p.m. and 8:15 p.m. and declined gradually until midnight (Video 1, Supplementary Fig. S1d). The number of crabs that moved from the sea to the land after releasing zoeae started to increase 30 min after sunsets and peaked between 9:15 p.m. and 9:30 p.m. The total number of moving crabs on roads increased after sunset until high tide and declined subsequently. The average time taken for crabs to cross the road was 2 min 32 s (n = 35).

### Roadkill and lunar phase

In this study, 739 carcasses were found in a 1.387 km section over a period of 31 days. Most of the crabs crossing the roads were females with eggs (99.6%). Most roadkilled carcasses, 703 out of 739 crabs, were also females (95%). As the number of crabs that traveled to release zoeae was high at the new and full moon, the number of roadkilled crabs was also high (Fig. 1, Supplementary Fig. S1). During the days around the half moon, as fewer crabs migrated, roadkilled crabs decreased, confirming that the lunar phase was synchronized with the number of roadkilled crabs.

### Effects of traffic volume on roadkill

The traffic volume itself does not have effect on roadkill (R^2^ = 0.06).The combination of the traffic volume and moon brightness ratio had a statistical power of 83% for the number of roadkilled crabs, and the following regression equation was obtained (Adjusted R^2^ = 0.83, F = 57.63, p = 0.0004). Each regression coefficient was significant at a level of 0.05. Through this model, the potential number of roadkilled crabs is estimated through the moon phase and traffic volume, and the potential number of roadkilled crabs can also be estimated when the traffic volume increased or decreased.

$$\text{ln}\left(\widehat{\text{Y}}\right)=-1.99+8.06{\text{X}}_{1}+0.05{\text{X}}_{2}\left({\text{X}}_{2}\ne 0\right), \widehat{\text{Y}} = 0 \left({\text{X}}_{2}=0\right),$$

$${\text{X}}_{1}=\left|\text{Moon brightness ratio}-0.5\right|,$$

$${\text{X}}_{2}=\text{ Traffic volume at night},$$

$$\text{Y}=(\text{Number of roadkilled crabs})/\text{km}.$$

### Landscape effects on roadkill

**Figure 2:**
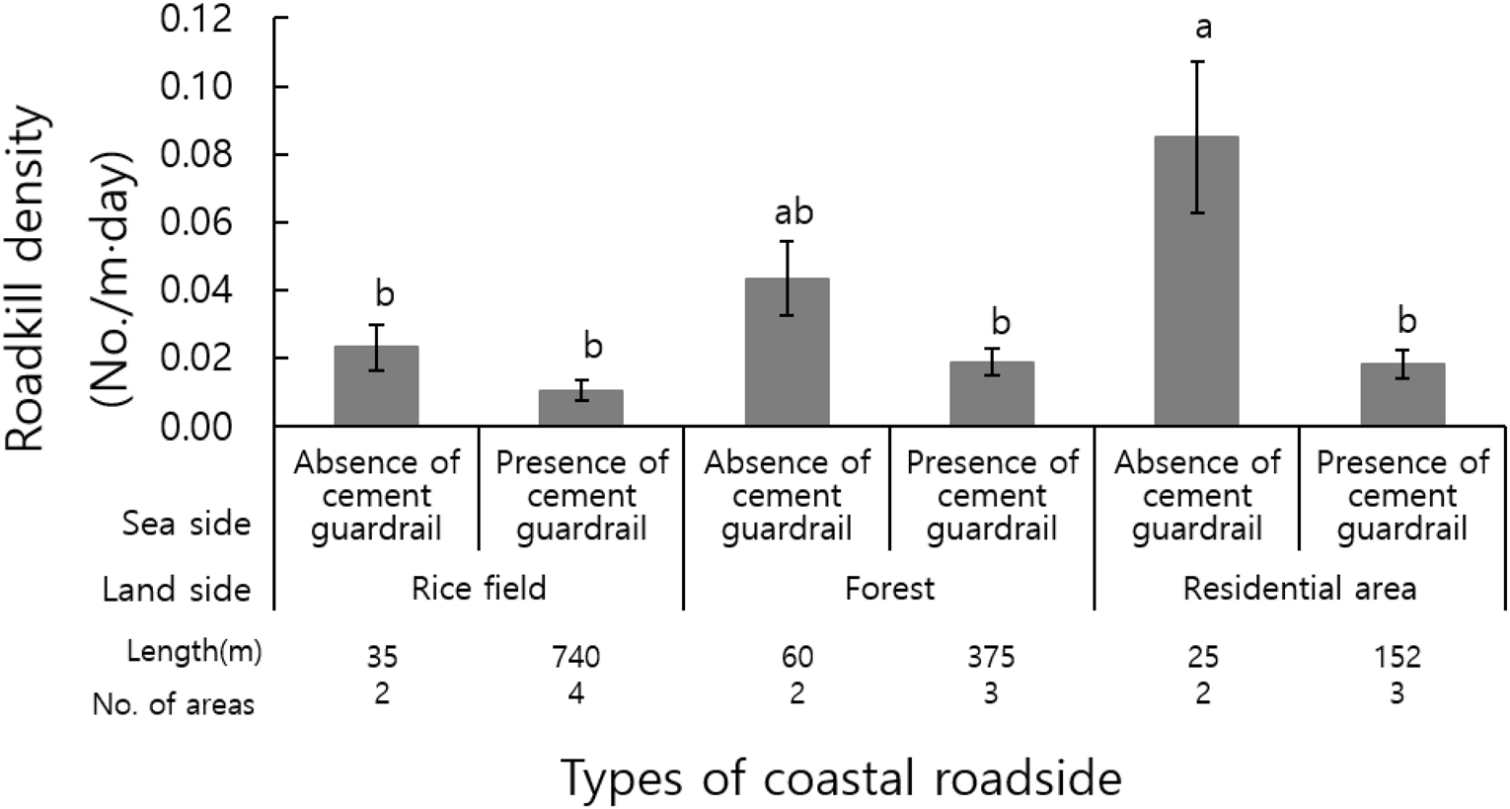
Roadkill density depends on the type of coastal roadside (ANOVA p < 0.001). Different letters denote a significant difference from other groups according to Scheffe’s post-hoc analysis, which suggests a difference between groups (p < 0.05).The vertical bars show standard errors for each group (n = 32).

The average of daily roadkill density on the land side was high in the residential area (0.052 crab/m day), followed by the forests (0.031 crab/m day) and rice fields (0.017 crab/m day). The roadkill density on the seaside was higher in the section without the cement guardrail (0.050 crab/m day) than the section with the cement guardrail (0.016 crab/m day) (Fig. 2, Table 1).

**Table 1:** Results of two-way analysis of variance (ANOVA) on the roadkill density.

	Landside		Seaside		Landside × seaside	
	F	*P*	F	*p*	F	*p*
Roadkill density (/m day)	5.260	0.0060	15.451	0.000	3.457	0.0335

In the post-hoc analysis using Scheffé’s method, the roadkill density was the highest in the section for the residential area without cement guardrails with the average difference from the other categories, except for the forest section without cement guardrails (Fig. 2, Table 1).

## Discussion

We found the breeding migration of land crabs led to high roadkill density. The majority of roadkilled crabs were ovigerous females crossing coastal road on the way to breeding near the seashore or returning. In some species, breeding migration contributed to frequent roadkill (e.g., amphibians^35^; brack land crab^18^). Especially, animals in which the adult and larval habitats are separated show high mortality on the roads bisecting them^9^.

### Effects of migration rhythm on roadkill

The breeding migration of *Sesarma haematocheir*in this study showed a synchronized rhythm related to the moon phase. The number of female land crabs releasing larvae peaked during the syzygy tides (new/full moon) and decreases around the half-moon phase. These results are in agreement with prior reports^26,39–43^. Not only the species in this study but a wide range of decapod crustaceans shows a rhythmic release of larvae relating to the lunar phase, light–dark, and tidal cycles^44–46^. Semilunar rhythms are most common among littoral and supralittoral species^44^ and estuarine decapod crustaceans^47^. Such rhythms of larval release are usually during nocturnal spring high tides at the new or full moon and it occurs most often in the first half of the night, so larvae have a better chance to survive^36,46^. Because releasing zoeae during nocturnal high tide reduces the risk of predation for both adult females and larvae^44,48^ and prevents larvae from reaching the higher levels of the estuary, where dangerous combinations of high temperature and low salinity^44,49^. Overall, the combination of release in the early evening and during high tides has an adaptive value^40,49^.

In this study, 739 carcasses were found along the 1.387 km stretch over 32 days, the majority (95%) of roadkilled crabs were mature females migrating to release the zoeae. The annual roadkill density can be estimated 1594 crabs/km year, which was very high compared with the roadkill density of other species. For example, the roadkill density of vertebrates in Columbia was 87.8/km year, with the roadkill density of snakes being 78.8/km year^50^. The mortality of black land crab (*Gecarcinus ruricola*) recorded 132/km night on San Andres in 1997 and 751/km night on Old Providence in 1996^18^. Overall, the roadkill density of land crabs in this study showed significantly high when compared with that of mammals, reptiles, amphibians, and birds reported in previous studies.

### Effect of traffic volume and landscape on roadkill

Traffic volume itself was not correlated with roadkill density (R^2^ = 0.06). However, the combination of the traffic volume and the moon brightness ratio had clearer relationship with roadkill density (R^2^ = 0.83) than the moon brightness ratio itself (R^2^ = 0.78).

There were more roadkilled females with eggs that have been killed on the way to the sea, and the rest may have been killed on the way back to their habitat after releasing zoeae. A possible explanation is that the traffic volume in the early night (5.8 unit/h) is higher than that in the late night (1.8 unit/h).In the previous studies, the roadkill density was found to increase as the traffic volume increased in various species^33,51,52^. Costa et al.^53^ showed that increased traffic volume also increased ghost crabs kills on the beach. Tsai et al.^54^ also reported that the roadkill of *Sesarma haematocheir* on a dike was correlated with traffic volume. Because migrations of land crabs are highly synchronized during a few hours around syzygy, traffic volume at syzygy may critically affect the roadkill density in a short migration time. Since the construction of coastal roads, female land crab roadkill has occurred during breeding migration. If the traffic volume increased over a long time, it would affect population model, the local land crab population itself will be decreased.

In this study, the highest roadkill density of land crab was recorded in the residential section without cement guardrail. The cement guardrail seemed to hinder the movement of land crabs, and the section without a cement guardrail was used as a moving passage for the crabs, and the section had an entrance to the holiday resort and a parking lot with more traffic. As coastal roads have been developed, not only vehicular traffic increased, but a holiday resort was developed, which changed the surrounding environment and increased the roadkill density.

The spatial patterns of roadkill have shown to vary depending on land usage, road types, location factors and lifecycle^32,33,55^. For example, the roadkill of reptiles and amphibians was concentrated on the waterside due to movements related to spawning, dispersion of offspring^36,38,56^. In case of ghost crab, areas with densest population nearby beaches have higher probability of crab roadkills despite of light pollution as a potential ecological trap^21^. Overall, animals moving between water and land show high roadkill density when the transition zones of the habitat are fragmented by roads. Thus, the coastal region has important implications as transition areas for the land crabs^56^.

### Roadkill effects on demography

The sex ratio of *Sesarma haematocheir* in Japan found fewer males than females during the active season^26^. However, in our study, female adult crabs were only 29.6% in the adult habitat. This result can be explained by the fact that female crabs were disproportionately roadkilled during breeding migration. Importantly, as the crab body size increased, the proportion of females decreased significantly^57^. However, sex ratios of adult land crab were not biased in uninhabited islands^57^. As a result, significant male-biased sex ratios of land crab in this study are attributed to female skewed road mortality during spawning migration.

The breeding migration of several species increases the risk of roadkill for females. The roadkilled freshwater turtles were found during the breeding season, most of which were sexually mature females using the causeway to nest^58,59^. Population demographics may shift rapidly when reproductive movement disproportionately exposes one sex to high road mortality^30,60^. In this way, the road continues to affect the surrounding animals over a long period of time.

### Conclusion: implications for roadkill mitigation

Female land crabs must migrate to the sea annually to release larvae, so they complete their lifecycle. Coastal roads act as significant barriers in this migration and increase road mortality. As strategies to mitigate land crab roadkill, underpasses, fences, or signs can be installed depending on the environmental characteristics of roads with a high roadkill density. However, land crabs have adapted to land life over a long time and can freely go up and down trees or cement retaining walls. Therefore, even though an underpass is installed, it is important to create fences using materials that prevent climbing by land crabs and manage them continuously.

It is important to understand the life history and behavior of animals to prevent animal deaths due to artificial structures including roads^54^. In the case of *Sesarma haematocheir*, collective migration to release zoeae rapidly synchronizes the roadkill risk. The key time of crab migration can be predicted based on the lunar phase. Therefore, if the traffic volume is controlled by predicting the key time in advance, such deaths among land crab can be prevented most effectively.

## Methods

### Study animals

*Sesarma haematocheir* is a land crab that inhabits terrestrial low lands or valleys^39^. After a winter hibernation, they mate from May to August, and the female crabs carry eggs for one month between June and September^26,40,41^. The ovigerous (egg-bearing) crabs mainly migrate to coasts or estuaries that flow into the sea to release larvae between sunset and midnight during the spring tide^36,40^. When female crabs arrive at the waterside, they open their abdominal flap and shake their body to release zoeae (Video 1). Hatching is highly synchronized, with many embryos hatching in 1 h^42^. The number of females releasing larvae is increased at new/full moon and decreased at half moon. Larvae of land crabs live in the sea and return to brackish water meadows to develop into young crabs. As they enter their third summer, they migrate to the habitat of adult crabs^26^.

### Study area

**Figure 3:**
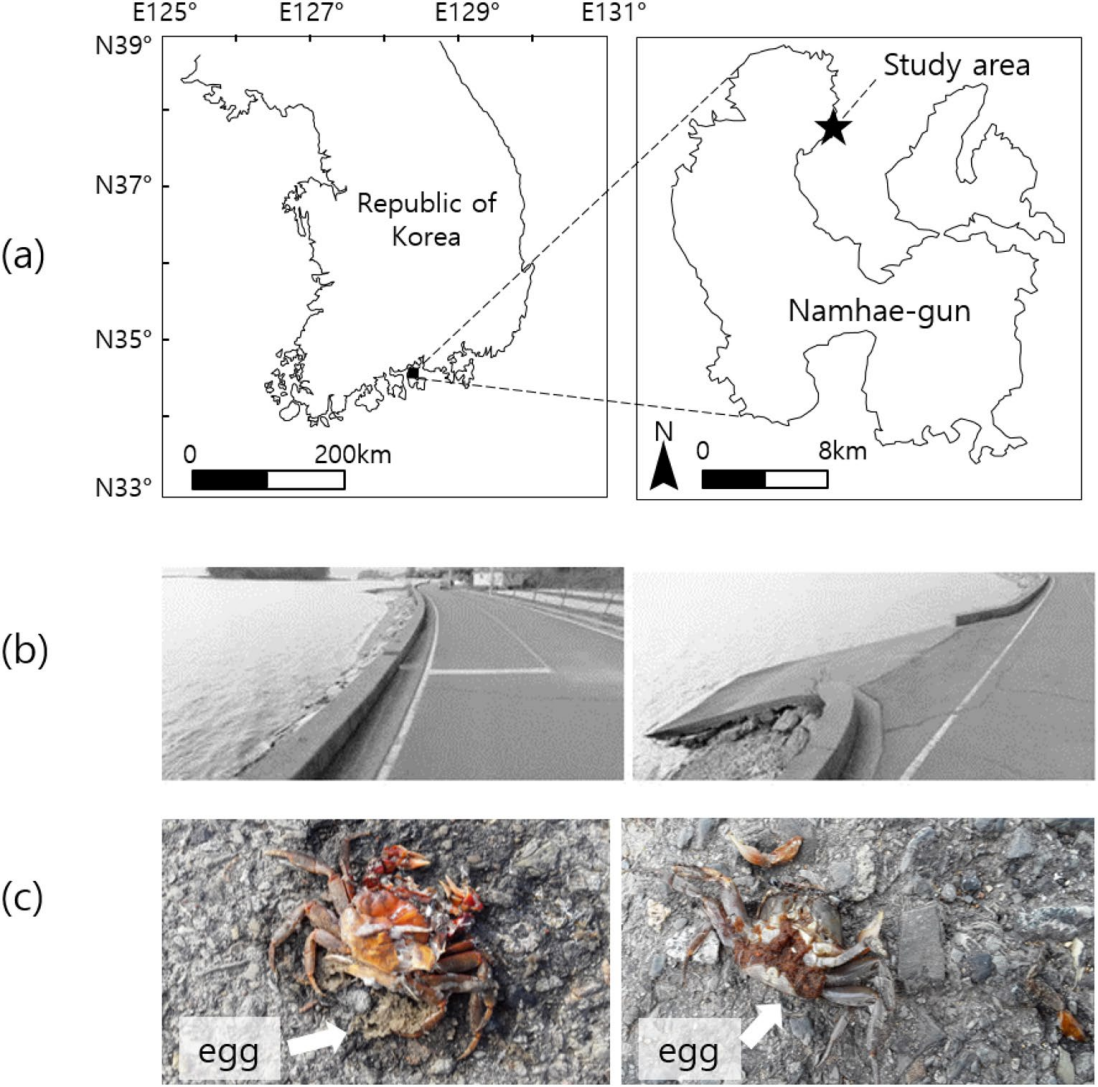
Study area. (**a**) Map of the study area, (**b**) Cement guardrail present and absent section on sea side, (**c**) roadkilled crab.

This study was conducted in Jinmok-ri, Seolcheon-myeon, Korea, in adult land crab habitat (Fig. 3a). The coastal roads were constructed in 2005. The land side of the coastal roads comprised rice fields and forests with scattered houses. The coastal roads on the land side was divided into rice fields, forest, and residential areas, while the environment on the sea side was divided whether or not a cement guardrail was present. The sea side of the roads has a 50 cm high cement guardrail, and partially not (Fig. 3b). The section without cement guardrail has a ramp to the sea for people to access to the intertidal area.

### Data collection

#### Crab migration

All experiments performed in this study complied with current laws of the Republic of Korea. The number of *Sesarma haematocheir* that migrated from land to sea and released zoeae was recorded from sunset to midnight^26^ for 31 days between July 28 and August 27, 2018. The section was an 11 m-long coastal roads with a forest on the land side and no guardrail.

#### Roadkill, traffic volume, the moon phase

We investigated the number of roadkilled crabs along the 1.387 km-long coastal road (Table 2, Fig. 3c). This section is neighbored with migration observing section (11 m). Every morning, between 5:30 a.m. and 7:30 a.m., were moved carcasses after recording to prevent duplication. The number of roadkilled crabs in each category was recorded including the number of females bearing eggs, the number of females without eggs, and the number of males.

**Table 2:** Roadkill observation area.

Sea side	Land side	Length (m)	No. of areas
Without cement guard rail	Rice fields	35	2
	Forests	60	2
	Residential areas	25	2
With cement guard rail	Rice fields	740	4
	Forests	375	3
	Residential areas	152	3
Total		1387	16

The volume of nocturnal traffic was measured for 24 days by installing a device carrying two light sensors connected to an Arduino Uno board on the road, which can be used to detect vehicle headlights. The device was programmed to automatically record the variance of light intensity at 1 s intervals. The traffic volume on this coastal road ranged from 12 to 34. The traffic was 30% more on the weekends. The traffic volume was 5.8 unit/h in early night, 1.8 unit/h in late night, 0.7 unit/h in pre-dawn. The speed limit is 60 km/h, however there are many curves on the road, so the car is usually driven at a lower speed.

We quantified the moon phase according to the brightness ratio, which is the proportion of bright area to the total area of the moonlight^61^.

#### Statistical analysis

To identify the effect of traffic volume on the release of zoeae and the number of roadkilled land crabs, a multiple regression analysis was conducted with the moon brightness ratio and traffic volume as independent variables using SPSS 25.0. The correlation of the moon phase and traffic volume is not significant (r = 0.11). Two way Analysis of variance (ANOVA) was also conducted using SPSS 25.0 software to investigate the roadkill density according to the environmental category on both sides of the road (n = 32).

## Supplementary Information

Supplementary Information 1.

Supplementary Information 2.

Supplementary Video 1.

## Data Availability

The datasets generated during and/or analyzed during the current study are available from the corresponding author on reasonable request.
