# Supplementary material for: Coastal road mortality of land crab during spawning migration
Source: Sci Rep. 2021 Mar 23;11:6702. doi: 10.1038/s41598-021-86143-z (PMC7988064; doi:10.1038/s41598-021-86143-z)
Supplement: Supplementary file 1 — Supplementary Information 1. [file 41598_2021_86143_MOESM1_ESM.docx]

**Supplementary information**

**Why do land crabs cross the road? Coastal road mortality of land crab during spawning migration**

**Mi Ryu^1^and Jae Geun Kim^1,2,3, *^**

^1^Graduate School of Interdisciplinary Program in Environmental Education, Seoul National University, Seoul08826, Korea

^2^Department of Biology Education, Seoul National University,Seoul08826, Korea

^3^Center for Education Research, Seoul National University, Seoul08826, Korea08826

^*^corresponding: jaegkim@snu.ac.kr

**
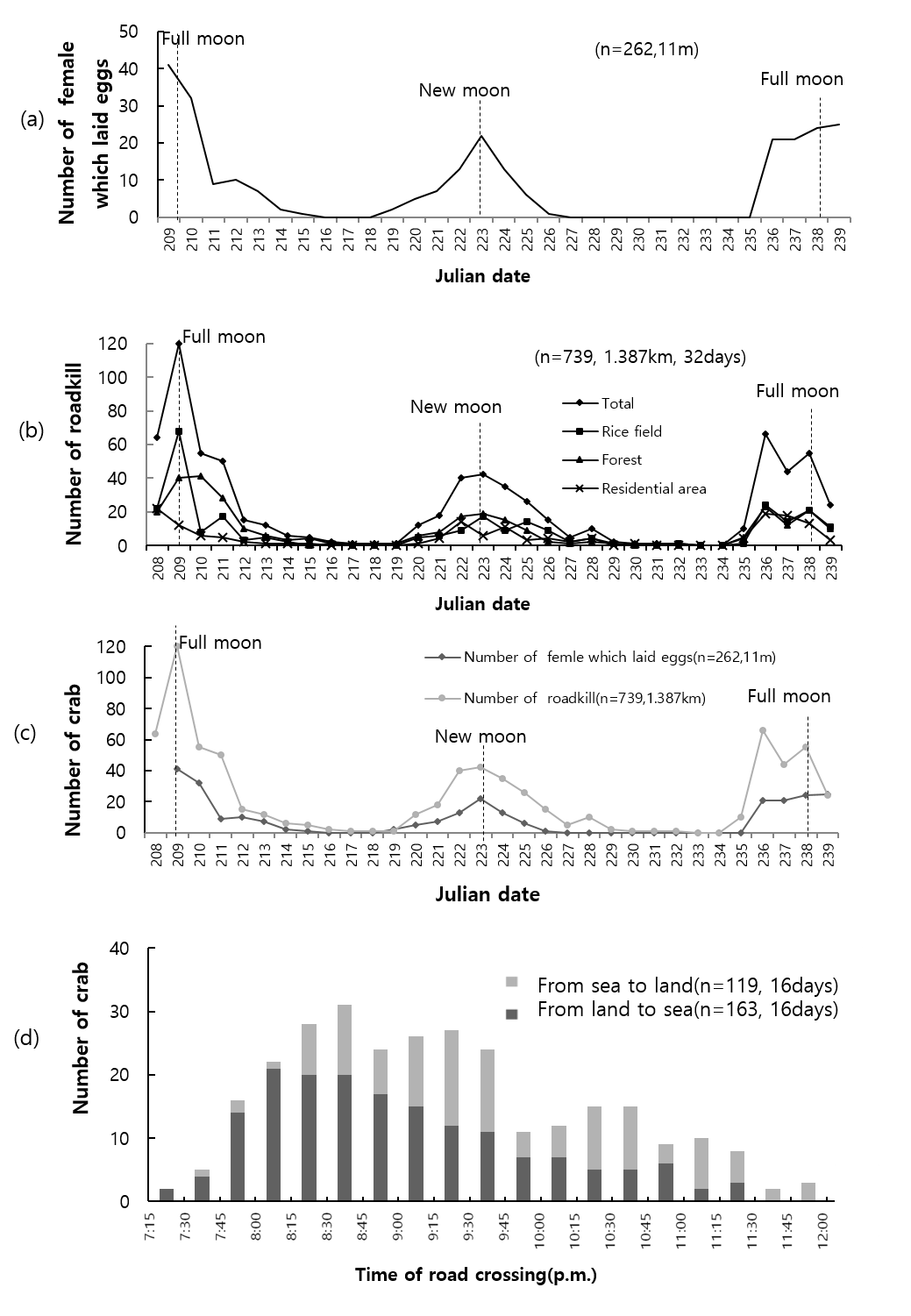
**

**Supplementary Figure 1**. Releasing zoeae and roadkill of *Sesarma haematocheir* (a) Number of female crabs that laid eggs each day, (b) Number of roadkilled crabs found each day, (c) Number of roadkilled crabs found each day (1.387 km) and number of females that laid eggs each day (11 m), (d) Number of crabs crossing the road per 15 min.
