# Supplementary material for: Coastal road mortality of land crab during spawning migration
Source: Sci Rep. 2021 Mar 23;11:6702. doi: 10.1038/s41598-021-86143-z (PMC7988064; doi:10.1038/s41598-021-86143-z)
Supplement: Supplementary file 2 — Supplementary Information 2. [file 41598_2021_86143_MOESM2_ESM.docx]

**Video 1**. Zoea release activity of land crab
